# Production possibility frontiers in phototroph:heterotroph symbioses: trade-offs in allocating fixed carbon pools and the challenges these alternatives present for understanding the acquisition of intracellular habitats

**DOI:** 10.3389/fmicb.2014.00357

**Published:** 2014-07-17

**Authors:** Malcolm S. Hill

**Affiliations:** Department of Biology, Gottwald Science Center, University of RichmondRichmond, VA, USA

**Keywords:** *Symbiodinium*, *Chlorella*, investment strategies, endocytobiology, intracellular mimicry, phagosomes

## Abstract

Intracellular habitats have been invaded by a remarkable diversity of organisms, and strategies employed to successfully reside in another species' cellular space are varied. Common selective pressures may be experienced in symbioses involving phototrophic symbionts and heterotrophic hosts. Here I refine and elaborate the Arrested Phagosome Hypothesis that proposes a mechanism that phototrophs use to gain access to their host's intracellular habitat. I employ the economic concept of production possibility frontiers (PPF) as a useful heuristic to clearly define the trade-offs that an intracellular phototroph is likely to face as it allocates photosynthetically-derived pools of energy. Fixed carbon can fuel basic metabolism/respiration, it can support mitotic division, or it can be translocated to the host. Excess photosynthate can be stored for future use. Thus, gross photosynthetic productivity can be divided among these four general categories, and natural selection will favor phenotypes that best match the demands presented to the symbiont by the host cellular habitat. The PPF highlights trade-offs that exist between investment in growth (i.e., mitosis) or residency (i.e., translocating material to the host). Insights gained from this perspective might help explain phenomena such as coral bleaching because deficits in photosynthetic production are likely to diminish a symbiont's ability to “afford” the costs of intracellular residency. I highlight deficits in our current understanding of host:symbiont interactions at the molecular, genetic, and cellular level, and I also discuss how semantic differences among scientists working with different symbiont systems may diminish the rate of increase in our understanding of phototrophic-based associations. I argue that adopting interdisciplinary (in this case, inter-symbiont-system) perspectives will lead to advances in our general understanding of the phototrophic symbiont's intracellular niche.

## Background

Symbiotic associations between different species with conjoined evolutionary trajectories are among the most common ecological interactions in biological communities (Thompson, 2005; Douglas, 2010). They also represent some of the most important evolutionary moments for life on this planet given that the genesis of the Domain Eukarya involved successful invasion of host cells by bacterial endosymbionts (e.g., mitochondria and chloroplasts—Knoll et al., 2006). Symbiotic interactions are exceptionally diverse and include everything from pollinators/mycorrhizal symbionts and their plant hosts, to parasites that castrate snails, to intracellular mutualists and parasites (e.g., Thompson, 2005; Douglas, 2010; Vergara et al., 2013). The evolutionary responses of endocytobiological associations are particularly interesting due to the high degree of intimacy between partners, which has the potential to generate complicated evolutionary patterns as the host and symbiont respond to the selective pressures each places on the other (e.g., Thompson, 2005).

Organisms that occupy intracellular habitats must avoid the host's cellular defenses (e.g., immunological response, phagotrophy—Scott et al., 2003; Martirosyan et al., 2011; Sibley, 2011). Despite the challenges of living inside a cell, many symbionts have successfully invaded this habitat as parasites and mutualists (e.g., Schwarz, 2008; Nowack and Melkonian, 2010; Heinekamp et al., 2013; Romano et al., 2013). The dynamic adaptive landscapes associated with endocytobiological interactions can generate tight integration between the partners such that the evolutionary manifestation is an obligate association for one or both species (e.g., Amann et al., 1997). From an evolutionary perspective, however, the earliest stages of intracellular occupancy must, to some degree, involve facultative associations. It is clear that we do not fully understand nuanced aspects of evolutionary processes that shape many intracellular interactions, and thus the patterns (e.g., host specialization; Thornhill et al., 2014) that emerge from them.

**Figure 1:**
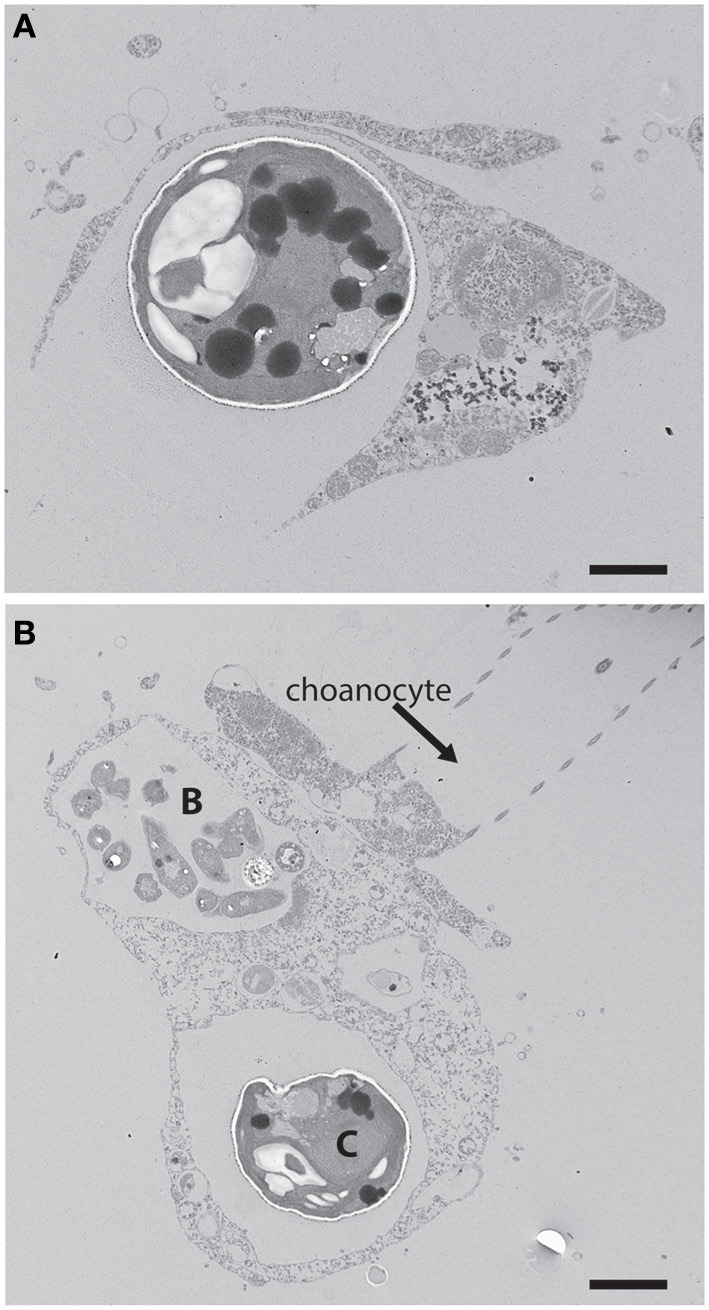
**(A)** An example of phagotrophic entry of a potential phototrophic symbiont into a heterotrophic host cell. *Chlorella* were fed to the freshwater sponge *Ephydatia muelleri* where they were captured through phagocytosis by archaeocytes. Scale bar = 1 μm. **(B)** *Chlorella* cell (C) and bacterial prey (B) within separate vacuoles of an archaeocyte adjacent a choanocyte in *E. muelleri*. The Arrested Phagosome Hypothesis states that the fate of some algal cells (i.e., symbionts) may differ from other potential prey items (e.g., bacterial prey in the archaeocyte) because algal symbionts can avoid digestion by translocating photosynthate to the host thus mimicking digesting prey (Hill and Hill, 2012). Scale bar = 2 μm.

Symbioses between phototrophs and heterotrophs are common in many ecosystems (e.g., lichens, *Chlorella*- and *Symbiodinium-*based symbioses). These ancient associations have been a focus of study for decades in a variety of systems (e.g., Karakashian and Karakashian, 1965; Kremer, 1980; Weis, 1980; Wilkerson, 1980; Brodo et al., 2001; Yuan et al., 2005). The algal partners often contribute substantial energy reserves to their hosts, and in many cases are located intracellularly. In some cases, adaptations for symbiotic life styles have been detected (e.g., Blanc et al., 2010). The dynamics of establishing the partnership from one generation to the next are complex, and depend upon characteristics of the species involved in the association. In many cases, algae re-infect hosts each generation from environmental sources. The route of entry into the host for intracellular partnerships is often phagotrophic (Figure 1), but the mechanisms that prevent activation of host defenses as a response to the foreign agent (i.e., symbiont) are often unknown. A common narrative can be found in much of the literature. Host cells lack particular vital nutrients, which they obtain from an endosymbiont. Through its beneficence (e.g., preferentially shutting down immunological or digestive processes in response to appropriate algal partners), the host creates a microhabitat, often within specialized cells, that favors algal growth, but only up to a point. If the symbiont population becomes too large, the host imposes some type of control to maintain symbiont population size near a carrying capacity. Under this scenario, hosts must coordinate a complicated choreography of genetic and cellular events in response to symbiont presence. In this context, algae play a limited role in this association, and some have gone so far as to liken them to prisoners involved in “enforced domestication” (Wooldridge, 2010; Damore and Gore, 2011).

Two hypotheses that afford symbionts a larger role in initiating and maintaining populations within host cells were presented recently (Hill and Hill, 2012). One of those hypotheses, the Arrested Phagosome Hypothesis (APH), proposes that phototrophs enter a host cell through phagocytosis (Figure 1). However, the APH states that the symbiont can then subvert normal endomembrane processes that lead to exocytosis by mimicking an organelle typically associated with digestion (e.g., the phagosome) through the perpetual release of photosynthetically-derived compounds. Thus, under the APH, symbionts have evolved a strategy involving the release of photosynthate so they may remain within the host cell (i.e., occupy habitat) for extended periods of time. It is important to note that the focus here will be on carbon-based photosynthate. This perspective builds on the work of biologists like Muscatine et al. (1981) who examined carbon contributions that zooxanthellae make to coral animal respiration. A major difference from that earlier work and the ideas presented here is that I will focus on strategies that the symbiont employs to procure its cellular habitat. It is also important to note that symbioses like the ones considered here involve nuanced and complicated host-to-symbiont and symbiont-to-host transactions of material like nitrogen and metals involved in photosynthesis (e.g., Fagoonee et al., 1999; Whitehead and Douglas, 2003; Pernice et al., 2012). While the perspectives presented should apply to any material exchanges between symbiont partners that involve trade-offs, the focus here will be on carbon alone. While the APH was proposed to explain how *Symbiodinium* procure residency within heterotrophic hosts in tropical habitats (Hill and Hill, 2012), the hypothesis should apply to nearly any phototroph:heterotroph symbiosis (e.g., those involving *Chlorella* or cyanobacteria, lichens).

Factors driving algae to occupy a host or host cell may differ depending on the partners and the habitat in which the symbiosis occurs. That is, these associations are likely context dependent. For example, selective pressures generated by the limitation of metals, which are essential for electron transport and can be rare in many environments, may be a factor favoring entry of phototrophs into the host cell habitat (Raven et al., 1999; Saenger et al., 2002; Rutherford and Faller, 2003). For example, in marine systems, the ratio of magnesium to calcium in modern seawater is approximately 5:1. However, this ratio has shifted as rates of continental spreading and terrestrial erosion have waxed and waned (Garrisson, 2007; Ries, 2010). The second hypothesis that affords symbionts a greater role in initiating and maintaining populations within host cells is the Magnesium Inhibition Hypothesis (MIH) that was proposed to explain why *Symbiodinium* seem to prefer hosts that modify CaCO_3_ solubilities (Hill and Hill, 2012). The MIH states that *Symbiodinium* favor hosts that have the ability to concentrate or release calcium ions, which would otherwise be limiting in the system. In other systems, different host-derived resources (e.g., other limiting metals like iron) might be targets for intracellular occupancy.

Regardless of explanatory hypotheses like the APH or MIH, translocation of photosynthate is ubiquitous in phototrophic symbioses. Greater attention needs to be focused on the conflict that likely exists between host and symbiont over the quantity and quality of material that is translocated. If hosts benefit from greater translocation and symbionts benefit from translocating less material or material of lesser energetic value, then the antagonism between partners might lead to partner specialization as selection favors strategies that mitigate the conflicts. Here, I argue that selection on symbiont-driven allocation strategies deserves greater attention, and recent methodological and theoretical advances offer interesting avenues for future research. My purpose is to provide a useful heuristic for considering selective pressures algal symbionts and their hosts may face in the context of translocation.

## Durable vs. consumable trade-offs & the production possibility frontier

Phototrophic organisms create fixed carbon stores through photosynthesis. The chemical energy (e.g., reduced sugars) is then used to power a variety of physiological functions including basal metabolism and growth/reproduction. Excess energy can be stored for future consumption. Phototrophic endosymbionts face additional debits against their energy budget in the form of material translocated to the host. The APH views the intracellular space as one that can be leased from a host (Hill and Hill, 2012; Figure 1). The “cost” of occupying the intracellular space is the fraction of photosynthetically-fixed C that is translocated, which has been reported to reach 95% for some species of corals and dinoflagellate endosymbionts (Falkowski et al., 1984; Muscatine and Weis, 1992; Yellowlees et al., 2008; Muller et al., 2009; Stambler, 2011). While there is little doubt that material is translocated to heterotrophic hosts, Davy et al. (2012) point out that many deficits exist in our current understanding of the quantity and type of material translocated to heterotrophic hosts. The 95% value quoted above is too general and imprecise to be of use for specific symbioses, and greater work is required to create a realistic picture of the material that moves between symbiotic partners. Nonetheless, the physiological characteristics of the host cell would set the price of the space, and the symbiont would have to “pay” at a particular rate and with particular expectations of materials released. It is important to note that in addition to costs required to occupy the endomembrane system, host cells would also have a unique molecular genetic milieu (e.g., immunological responses) that would impose another level of selection on an invading symbiont. However, the APH points to a clear life history trade-off from the symbiont's perspective—for every increase in material translocated to the host, the symbiont suffers a reduction in the amount of energy available for other physiological needs like mitosis.

**Figure 2:**
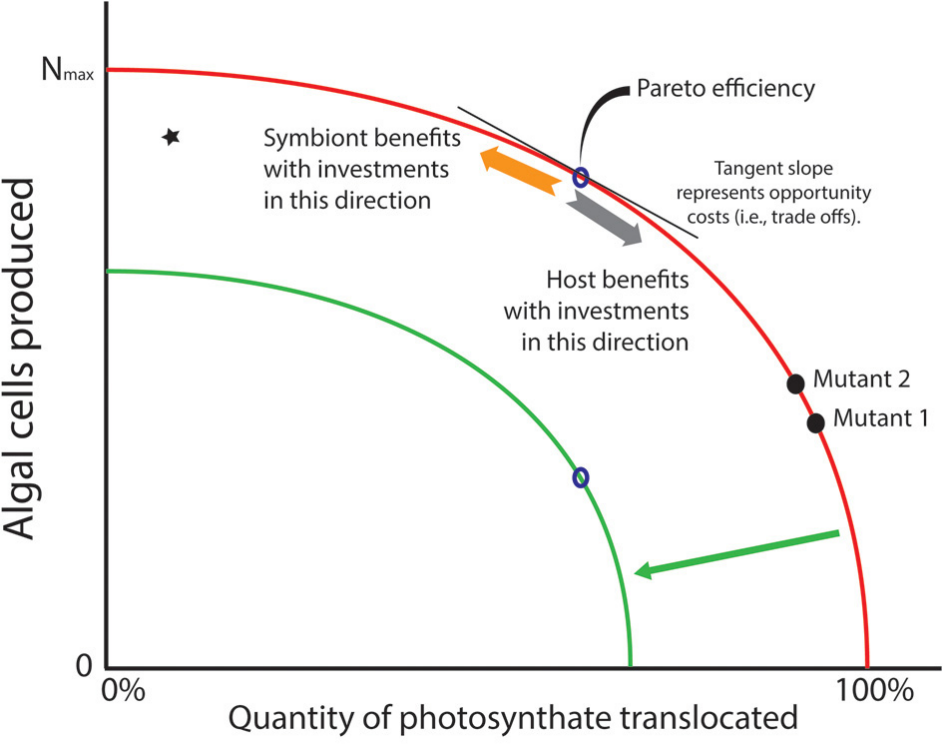
**Production possibility frontier (PPF; red curve) represents trade-offs in investment strategies that phototrophic symbionts may face with the photosynthate they create *in hospite***. Algae may use their energy stores to create more cells through mitosis (a durable good—see orange arrow), but this comes at the cost of carbon that is translocated to the host (a consumable good—see gray arrow). It is assumed that natural selection would rapidly remove inefficiencies (star in graph) where more carbon could be translocated or its energetic equivalents used for cell division. Thus, “Pareto efficiencies” that comprise the curve represent evolutionary optima. The tangent to the curve represents opportunity costs associated with producing one commodity over the other. A prediction of the Arrested Phagosome Hypothesis is that symbionts will increase the time they reside in a cell by translocating more material to the host (moving from mutant 2 to 1). However, if a mutant can release less photosynthate without losing its ability to evade host defenses (moving from mutant 1 to 2), then natural selection may favor that strategy as more cells will be available to colonize additional cells and hosts in the environment. If the PPF shifts inward (green curve) due to some major environmental event (e.g., thermal stress), the symbionts are faced with a smaller energy budget. If amount of photosynthate that must be translocated to meet host demands does not change, fewer cells can be produced (see open points on the red and green curves). This is a scenario that might lead to phenomena like coral bleaching.

For many of the algae that form symbioses with heterotrophic hosts, asexual reproduction is the dominant mode of population increase (Pettay et al., 2011; Thornhill et al., 2013), and mitosis is an energy consuming process as DNA, cellular machinery, organelles, etc. are duplicated to provision each daughter cell. Given that cell division and translocation draw on the same primary production pool of fixed carbon (ignoring for the moment basal metabolism and storage), investing in one or the other process raises the possibility that competition ensues for the energy represented by the limited products of photosynthesis. A common graph used in economics provides a useful tool to visualize the trade-off that phototrophic symbionts face (Figure 2). The production-possibility frontier (PPF; Gillespie, 2007) is a curve that depicts possible production sets representing the most efficient distribution of two commodities that draw on the same inputs for their production. Points on the curve are known as “Pareto” efficiencies (Gillespie, 2007). The curve also helps define the opportunity costs that exist within a system. A typical example from economics highlights the trade-offs that exist when an agent can decide whether it should produce a durable or a non-durable (i.e., consumable) good. Durable and non-durable goods are often compared in this manner because shifts along the PPF provide information about the investor's “interpretation” of future benefits of current investments. That is, investing in a durable good provides some indication that the investor interprets conditions as conducive for future growth. For algal symbioses, we can consider two commodities in which an algal cell might invest. The first is production of new cells generated through mitosis—new algal cells are analogous to durable goods since they last for a substantial period of time. The second way that energy might be invested involves translocating photosynthate to the host—this is analogous to investing in a non-durable/consumable good that is used up immediately. As with any trade-off, investment in one commodity necessitates reducing investment in the other. In phototrophic symbioses, the PPF represents the set of ratios of cells produced relative to material translocated to the host. Each point along that curve represents the most efficient number of algal cells produced for that amount of fixed carbon translocated to the host (Figure 2).

The only way to increase the number of algal cells produced for a particular investment in translocated material is to shift the PPF outward. But moving the PPF requires a change in the pool of fixed carbon that is available for investment (e.g., an algal mutant that is more photosynthetically efficient, or the environment changes so light levels or nutrient load is higher). An inward shift of the PPF represents a scenario where the pool of carbon available for investment decreases, which might be expected when photosynthetic ability is compromised (e.g., under thermal stress). Under this scenario, only symbionts that could maintain a level of translocation to meet host demands, and establish a rate of population growth that was sustainable, would remain in symbiosis. If we assume, however, that the system is static (i.e., no improvements in technological (i.e., physiological) abilities to increase the fixed carbon pool), then natural selection could act on strategies that algal symbionts employ to gain competitive advantages within a particular host. For example, a mutation that gives its bearer elevated cell division rates (and thus lower translocation rates, mutant 2 vs. mutant 1 in Figure 2) might appear in a symbiont population harbored by a single host. Provided that this mutant does not trigger a defensive or digestive response from the host, it would have a competitive advantage over other individuals in the symbiont population (see Frank, 1996). This opens the possibility of evolutionary changes within hosts, and possibly among the other hosts that exist in the habitat (but see Damore and Gore, 2011). Alternatively, if residence time is the phenotype that natural selection favors, a mutant that translocates more fixed carbon (with lower rates of division) might increase in frequency because the host detects it less frequently (e.g., higher translocation rates of mutant 1 vs. mutant 2 in Figure 2).

But what evidence exists that phototrophs face the kind of trade-off in translocation vs. mitosis envisioned here? There are many indirect lines of evidence that a trade-off exists. It has long been known that algae translocate carbon and that the dynamics of that translocation process are complicated. For example, almost a half-century ago, Smith et al. (1969) consolidated evidence that metabolite transfer from symbiont to host is a wide-spread phenomenon in mutualistic and parasitic associations. It has also been known for many years that cultured *Symbiodinium* release only a fraction of their photosynthate compared to algae found in hosts; cultured algae also have distinct morphologies compared to algae in intact symbioses (e.g., Colley and Trench, 1983; Domotor and D'Elia, 1986). Ritchie et al. (1997) found that a commercially available synthetic fungicide stimulated release of fixed carbon products. More recently, Grant et al. (2006) found that a host release factor from the coral *Plesiastrea versipora* stimulated the release of glycerol from its *Symbiodinium* symbiont. The authors argued that the diversion of glycerol from the algae reduced internal stores of triacylglycerols and starch, which in turn would help the host regulate growth of intracellular algae. However, Suescún-Bolívar et al. (2012) provide the most direct test of the existence of trade-offs in phototroph:heterotroph symbioses. They induced release of glycerol from *Symbiodinium* growing in culture by exposing the dinoflagellates to osmotic up-shocks. The osmotic treatments did not affect photosystem performance or survivorship, but did reduce population sizes, which the authors attributed to a reduction in cell division rates for the *Symbiodinium* that released glycerol. These results should be interpreted carefully given that glycerol released by *Symbiodinium* may be a response to stress and not a translocated compound (see review by Davy et al., 2012).

Despite the caveats mentioned previously, there is evidence that the type of trade-off envisioned in the PPF (Figure 2) exists in phototroph:heterotroph symbioses. Furthermore, there seems to be a significant capacity for modifying the quality and quantity of material translocated. Burriesci et al. (2012) found that highly-efficient mechanisms exist for translocation of newly synthesized glucose from *Symbiodinium* to its *Aiptasia* host. Glucose appeared in host tissue as quickly as 2 min after exposing anemones to stable isotopes of CO_2_ and moving them into the light after rearing them in the dark. Other solutes appeared in host tissues at much later time points. The solutes mannose, inositol, threonine, glutamine, and succinate appeared after 1 h. Other solutes appeared after 1 day (e.g., glycerol, glutamic acid, and pentaric acid) and 1 week (e.g., glycine and ß-alanine), though these compounds may represent downstream products of host metabolism (e.g., Starzak et al., 2014). If variability in the release rates of these and other compounds exists among phototrophs within a population of symbionts, then natural selection could operate to favor variants with strategies that optimally match the characteristics of the host cellular machinery—a process that might ultimately lead to host specialization (Thornhill et al., 2014).

One of the most important insights gained from the PPF perspective is a clear statement of the problem of conflicts between partners in phototroph:heterotroph symbioses. As far as translocated carbon is concerned, hosts would appear to favor symbionts that give up more material because host fitness would increase. Symbionts, on the other hand, would appear to favor hosts that demand fewer of their photosynthetically-derived reserves because they could translate those energy gains into additional mitotic events (enhancing within- and among-host competitiveness). The phenotype observed in a particular holobiont combination is thus the manifestation of a tug-of-war between the competing pressures of translocating more material [to reduce the probability of being detected by the host, thus increasing within-cell residence time] and dividing more rapidly [which would produce more cells and might confer a competitive advantage through higher infective/dispersive capabilities compared to slower growing mutants]. It seems that productive research possibilities exist in exploring the precise mechanisms that regulate the evolutionary and ecological interactions between partners in the context of these tradeoffs.

Furthermore, by emphasizing the reciprocal dynamics of host:symbiont interactions in terms of material goods that are exchanged between partners (as the APH does—Hill and Hill, 2012), an opportunity exists to consider one mechanism that might lead to specialization between partners. There are likely many strategies available to symbionts that would lead to faster growth rates (see above), and selection would often favor faster growing symbionts that remain undetected by the host. The symbionts have short generation times, large population sizes (albeit small effective population size due to clonality), and mutation rates—conditions that would provide constant fuel for rapid evolutionary change. The relative fitness of different symbiont strains (created via mutation) constitutes a major force that might drive rapid lineage turnover within a host. The long-term fate of these common genetic changes would depend on the interplay of effective population size and natural selection. Population-level processes such as selection, migration, and recombination will also help shape the genetic diversity of symbionts among and within hosts (Santos et al., 2003; Thornhill et al., 2009, 2013; Andras et al., 2011; Pettay et al., 2011). The genetic footprint of these processes is likely to be complex, but it is clear that opportunities exist for rapid adaptation of symbiont to its host environment. Within each host, symbiont populations might experience diversifying selection driven by pressure to evade immune systems (e.g., Endo et al., 1996) while simultaneously experiencing stabilizing or directional selection in response to the host's energetic expectations for translocated material. Rapid onset of local adaptation by the symbiont to its host (involving selective sweeps) might be expected (e.g., Thornhill et al., 2014).

Contrary to the perspective presented above, some theoretical models find that symbionts become enslaved partners precisely due to the substantial differences in evolutionary rates between partners (e.g., Frean and Abraham, 2004; Damore and Gore, 2011). In these models, the host does not respond to the selective pressures created by the symbiont because its relative evolutionary rate is so much slower than the symbiont's. The rapidly evolving species, typically the symbiont, becomes highly cooperative, while the slowly evolving one, typically the host, does not reciprocate the cooperativeness (Frean and Abraham, 2004; Damore and Gore, 2011). However, these models often assume a quality of interaction (especially from the perspective of the symbiont) that is difficult to defend from biological first principles.

Many are beginning to explore the role of the symbiont in processes of invasion and establishment of intracellular residency, and as agents with independent evolutionary trajectories (e.g., Rodriguez-Lanetty et al., 2006a; Schwarz, 2008; Weis et al., 2008; Davy et al., 2012), but a host-centric lens is still too often applied to understanding the associations in ways that mask possible important interactions. Much of the difficulty of studying these microscopic symbionts is due to their opaque life histories. One overgeneralizes if host:symbiont interactions involving *Symbiodinium*, *Rhizobium*, *Buchnera*, endomycorrhizae, or phages infecting cyanobacteria are lumped together as if they behave identically - as has been implied in some of the models developed to date (e.g., Frean and Abraham, 2004). These associations involve quite different agents of biological interaction operating at different scales and degrees of intimacy. While the models that have been developed describe interesting dynamics, biologists must determine to which symbioses they apply. For example, Frean and Abraham (2004) state that “Surprisingly, in very few cases have endosymbionts been shown to benefit significantly from their interactions with host organisms….For the putative benefits of symbiotic life as zooxanthellae, dinoflagellates give up their cell wall and their flagella, sacrifice most of their photosynthetic products, and reduce their reproductive rate.” Rather than viewing these as losses, it may be more profitable to look at them as strategies for host occupancy and for production of daughter cells to infect new hosts. In some models, the symbionts are assumed to enter a host where they become trapped until the host dies (Frean and Abraham, 2004). This assumption can be rejected for many of the algal symbioses that create stable phototroph population sizes despite constant input from mitotic events, which indicates that each algal cell has a particular residence time within the host and a free-living stage in the environment (Hill and Hill, 2012). Further, the payoff matrices used in some approaches that employ game theory (e.g., the snow drift model—Damore and Gore, 2011) do not map on to or reflect symbioses we find in nature. There are reasons to believe the payoffs experienced by the hosts and symbionts operate on different scales with different magnitudes.

Cooperation and defection might be appropriate terms to describe some interactions (e.g., production of a joint nutrient), but they fail to describe the types of interactions that occur if cellular mimicry is in play, as has been proposed in the APH (Hill and Hill, 2012). That is, how can a host cooperate if it is “unaware” that it is in a game, and the two species are not “fighting” over the benefits of a mutualism? If a host is being duped by a symbiont, the situation described in some theoretical approaches begins to dramatically violate assumptions of the model, which calls in to question the generality of the findings (e.g., Damore and Gore, 2011). In this light, the co-evolutionary possibilities become more intriguing, and additional model approaches may be beneficial (see also Frank, 1996; Friesen and Jones, 2012). For example, it may be that modern scleractinian corals are ecologically naïve, and have evolved reduced predatory efficiency because they have been energetically subsidized for millions of years by their *Symbiodinium* symbionts. Plasticity in host feeding, and a strong feed-back system between symbiont and host, indicate a continued reliance on heterotrophy by both the host and symbiont (e.g., Grottoli et al., 2006; Ferrier-Pages et al., 2010). However, the assurance of energetic inputs from algae, extrapolated over many millennia, may have weakened the selective pressure on structures and behaviors involved in predation. Contrary to the models described above, it may be that coral hosts have been selected to be, in a sense, highly cooperative.

## Dynamic energy budget perspectives and mechanisms of host:symbiont interaction

The trade-offs articulated above emphasize identifying optimal investment strategies that a symbiont might adopt to reside within heterotroph cells. Another useful perspective is one that looks at the consequences of changing the productive capacity of phototrophs (i.e., shifting the PPF). If efficient strategies exist for persisting in host habitats, then any stressors that decrease the productive capacity of the system would lead to major consequences for the holobiont. The green curve in Figure 2 represents a scenario where the fixed carbon pool available for investments has diminished greatly. If the intracellular residency costs remain the same, that is, the quantity of photosynthate required by the host stays at a certain level, then the number of cells that could be produced would drop. This is illustrated in Figure 2 where the open points on both curves are at the same location on the x- but not the y-axis. This might lead to reductions of the total number of symbionts harbored by the host, as observed in bleaching events for hosts with *Symbiodinium* symbionts.

**Figure 3:**
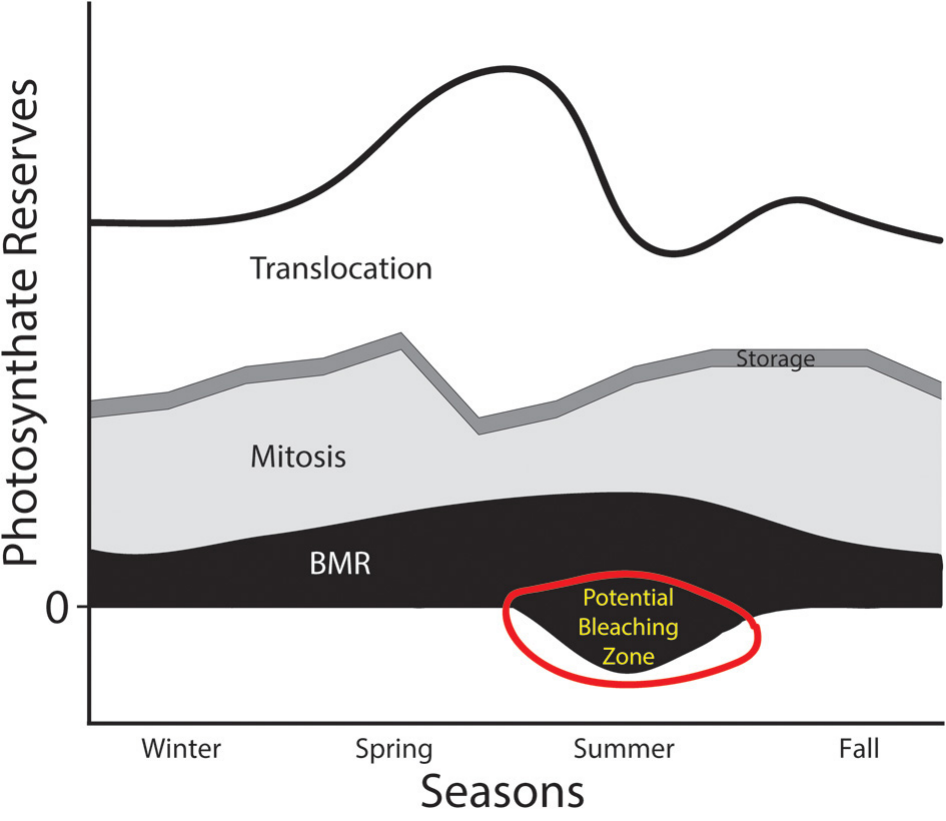
**Hypothesized annual photosynthate budget for an algal symbiont like *Symbiodinium***. The thick black line represents the total pool of photosynthate generated through carbon fixation. Four physiological compartments that energy derived from those photosynthates could be invested in include: (1) translocation to host (white), (2) storage (e.g., in lipids—thin dark gray band), (3) mitosis (light gray), and (4) basal metabolic rate (BMR in black). This figure envisions a drastic reduction in primary productive capabilities of the phototroph in the summer months (i.e., an inward shift of the PPF from Figure 2). This might be caused, for example, by drastically warmer water. A reduction in the photosynthate reserves might push the symbiont into territory representing energy deficits, which might lead to detection, digestion, or expulsion by the host.

A situation where the PPF curve would be shifted inward might occur when environmental conditions change. For example, we can consider the dynamics of host:symbiont interactions in the context of seasonal changes in habitat (Figure 3). During the majority of the year, the symbiont can produce a sufficient amount of photosynthate to placate host demands and take care of its other physiological functions. As envisioned in Figure 3, the symbiont has some plasticity in its investment in different compartments, and might invest in more mitosis when the host's metabolic rates are low (e.g., in the winter). However, in some seasons costs associated with intracellular occupancy might increase (e.g., as the metabolic demands of the hosts and symbionts increase), which would necessitate consuming more of the total available photosynthate reserve in the service of translocation or basal metabolism. If the thermal stress continues to a point that compromises photosynthetic capability (e.g., PSII damage, Warner et al., 1999), then the amount of primary production that can be invested dwindles, and the energy budget can go into deficit territory. Viewed in this manner, periods of thermal stress that compromise a phototroph's ability to maintain the rate of fixed carbon transfer would elevate detection or expulsion rates. If that stressor persists, phenomena like coral bleaching might be the result (“potential bleaching zone” in Figure 3).

Using an energetic budget approach offers important opportunities to examine these symbioses (Lesser, 2013). For example, Muller et al. (2009) used dynamic energy budgets (DEB) to model flows of matter and energy between partners in a phototroph:heterotroph symbiosis. The authors made several simplifying assumptions including that only excess material (photosynthate or nutrients) are transferred between partners. With the DEB, Muller et al. (2009) found that ambient food density, inorganic nitrogen, and irradiance had little affect on symbiont density whereas light deprivation and nitrogen enrichment caused increases in density. The importance of this type of work is the attempt to compartmentalize physiologically-important processes so that nuanced insights might be gained about the nature of the interaction between partners. However, it is important to keep front-and-center the assumptions that these various approaches make—in particular careful consideration of how we describe energy equivalents (Lesser, 2013). Furthermore, several recent studies have measured and modeled the flow of material and energy in coral:*Symbiodinium* symbioses (Tremblay et al., 2012; Gustafsson et al., 2013, 2014). These studies provide detailed perspectives on the dynamism of exchanges that likely occur between partners.

To fully explore any dynamism of energy allocation and the trade-offs proposed above, we require precise information about the molecular and biochemical interactions that occur between the partners. Next generation sequencing provides opportunities to gain a nuanced and detailed understanding of the interactions that occur between partners at the finest levels of molecular, genetic, and cellular interaction. Recent advances in transcriptomic, proteomic, and metabolomic analyses offer tools to gain fine-scale molecular genetic perspectives on the physiologically-important processes mentioned above (e.g., Meyer and Weis, 2012). While bioinformatic tools will expand research opportunities, we also need classic physiological experiments that elucidate meaningful aspects of host:symbiont interactions. Recent work with stable isotopes highlight the promise of precisely documenting the material that is translocated from the symbiont to the host, and that is taken up by the host from the symbiont (Hughes et al., 2010; Weisz et al., 2010; Burriesci et al., 2012; Pernice et al., 2012). Furthermore, the development of aposymbiotic model systems provides a number of empirical possibilities to determine how different symbiont types modulate the relationship with a particular host (e.g., Hambleton et al., 2014; Riesgo et al., 2014).

Efforts to create “model” systems of study will expand empirical opportunities (e.g., Weis et al., 2008; Lehnert et al., 2012), but it is clear that we will gain much if we maintain an explicitly comparative approach to work on these intracellular symbioses. Indeed, adopting an explicitly comparative perspective that unites the findings from different symbiotic partnerships may elucidate common pathways to intracellularlity (see below). The consequences extend beyond the phototrophic mutualisms considered here as any symplesiomorphies identified may be equally valuable for studies of intracellular parasitisms (e.g., malaria, toxoplasmosis). Do parasites release material to secure intracellular habitats in a manner that shares similarities with what we see in phototrophic symbioses? What reciprocal changes might be found in host endomembrane proteins common to associations that involve phototrophs or parasites? What modes of parasite invasion apply to phototrophic associations?

While understanding the mechanisms of interaction at the cellular level are vital, the evolutionary behavior of these associations is relatively unexplored from theoretical perspectives. If the trade-offs described above (Figure 2) are important, the specific factors that contribute to particular strategies of persistence within a single host and within a population of hosts need elucidation—especially in the context of holobiont performance. The reciprocal selective pressures that host and symbiont place on each other create interesting evolutionary possibilities. How does specialization evolve in these systems, and do they behave like host:parasite systems that engage in time-lagged, frequency dependent interactions? Modeling these symbioses from metapopulation perspectives would be particularly interesting. Hosts represent habitat. These habitats have extinction rates that depend on the life history of the host species. For symbioses that involve horizontal-acquisition, habitats become available when aposymbiotic propagules appear in the environment. The within host population may be an asexually derived clonal population, but it is part of a larger metapopulation. Secord (2001) appears to be the first to appreciate this fact.

## Terminological differences: cautionary typological tales

“The beginning of wisdom is to call things by their proper name.” *Confucius*

“… [the one] who first seizes the word imposes reality on the other”

*T. Szasz*

It is likely that the trade-offs articulated above apply to any symbiosis that involves a heterotrophic host that harbors a phototrophic symbiont. However, terminological difference among fields compromises our ability to identify common strategies that might exist. For example, in *Paramecium*:*Chlorella* symbioses, algae are located within a perialgal vacuole derived from the host digestive vacuole (Kodama and Fujishima, 2010). In *Hydra*, *Chlorella* populate the perisymbiont space in digestive gastrodermal myoepithelial cells (Rands et al., 1992). In some sponges, specialized cells, termed “cyanocytes,” harbor large aggregates of cyanobacteria; other sponge hosts harbor cyanobacteria in digestive vacuoles (Wilkinson, 1978). In non-phototrophic symbioses, e.g., *Trypanosoma* parasitisms, the parasite may briefly reside in acidic parasitophorous vacuoles (Lu et al., 1998; Chen et al., 2008). If the symbiosis under study involves *Symbiodinium*, the dinoflagellate symbiont is harbored within the symbiosome (e.g., Roth et al., 1988). It is possible that these differently named structures share common origins.

Hinde and Trautman (2002) argued for the primacy of the term symbiosome when describing membrane-bound symbionts living intracellularly. However, the symbiosome, if it is a distinct component of the cellular machinery, is a derived trait, and I contend that focusing on the symplesiomorphic traits of the endomembrane system of heterotrophic hosts is a better approach to understanding the shared evolutionary and ecological pressures phototrophs face as they invade heterotrophic host cells. We assume much about biochemical and physiological differences between symbiont-bearing and “normal” endomembrane structures when we erect terms for the former (e.g., “symbiosome”). A useful starting point is to accurately describe the endomembrane system (i.e., the habitat as seen by the invading symbiont) typically present in host cells (see e.g., Kodama and Fujishima, 2010). We stand to learn more about the nature of the association if we understand the endosomal compartments a symbiont targets, and whether the symbiont-bearing structure retains characteristics of the original endomembrane structure. For example, might phototrophs maintain residency within a host cell by mimicking digesting prey via the phagosomal compartments (as hypothesized in Hill and Hill, 2012)? What subtle differences in the chemical characteristics of a membrane can a symbiont modify to appear to the host cell like a particular cell constituent (e.g., late endosome or phagosome) to create habitat space that is stable, persistent, and safe? These guiding questions are not new, and were prominent in earlier work on intracellular symbioses (e.g., Muscatine and Lenhoff, 1963; Trench, 1971; Karakashian and Karakashian, 1973; Karakashian and Rudzinska, 1981; Reisser et al., 1982).

The endomembrane system existed before the symbiosis, and thus understanding “normal” cellular processes will yield major insights in the diverse phototrophic:heterotrophic symbioses that exist on the planet. Despite very similar research objectives and approaches, the different fields can operate in semi-separate circles; for example, it is rare to find citations of the seminal work of, for example, Karakashian (Karakashian and Karakashian, 1973; Karakashian, 1975; Karakashian and Rudzinska, 1981) in publications focused on *Symbiodinium* symbioses, or Trench citations (Trench, 1971; Trench et al., 1981; Colley and Trench, 1983, 1985; Fitt and Trench, 1983; Trench, 1987) in *Chlorella*-based symbiotic research. Muscatine recognized the importance of taking advantage of the methodological tractability of one system (e.g., *Hydra*) to inform the other (e.g., *Symbiodinium*), and appreciated the major lessons that could be learned by paying attention to the findings from different systems (e.g., Hoegh-Guldberg et al., 2007). Davy et al. (2012) recently discussed the importance of comparative work done with different symbiont systems, and provided a review of the contributions of earlier biologists who combined insights from these different systems.

The endocytobiological structures important in intracellular symbioses likely share important biochemical features (i.e., important symplesiomorphies exist), and phototrophic symbionts (perhaps even some non-photosynthesizing parasites like *Trypanosoma* or *Plasmodium*) may co-opt cellular machinery using similar strategies as they invade eukaryotic cells (Schwarz, 2008). For example, Boulais et al. (2010) compared the proteomes of 39 taxa (from amoebas to mice), and identified an ancient core of phagosomal proteins primarily involved in phagotrophy and innate immunity. Looking for similarities between and among cellular habitats by different symbionts may offer important clues about universal processes that favor invasion of heterotrophic host cells. Recent work elucidating the detailed machinations of the phagosome at fine-scale levels of molecular and genetic resolution (Stuart et al., 2007; Stuart and Ezekowitz, 2008; Trost et al., 2009) highlights the opportunities to shed significant light on intracellular symbioses between phototrophs and heterotrophs.

Parasitophorous vacuoles, symbiosomes, and digestive vacuoles may share similar characteristics because they all target the normal endomembrane process of heterotrophic cells. Insights into diseases like malaria might come from a detailed comparison of the cellular processes operating in mutualisms involving algae. For example, Kuo et al. (2010)'s finding that the proteins GP2 and Niemann-Pick type C2 are upregulated in symbiont containing *Aiptasia* is intriguing given the role of these genes in modulating immune responses and lysosomal cholesterol transport (Kuo et al., 2010; Werner et al., 2012, respectively). Chen et al. (2004) found that a Rab protein, which is normally a regulator of endocytotic recycling, is recruited to phagosomes containing heat-killed, but not live, *Symbiodinium* introduced to *Aiptasia* hosts. This points to specific molecular genetic pathways (especially the Rab pathway) that permit successful colonization of host habitats by *Symbiodinium* since the symbiont may arrest one of the endomembrane structures in the phagosome position. Similar processes operate for *Trypanosoma* and *Plasmodium* parasitisms (e.g., Batista et al., 2006; Seixas et al., 2012). Several more recent studies have employed transcriptomic and proteomic methods to provide detailed molecular genetic perspectives on the host:symbiont interface (e.g., Rodriguez-Lanetty et al., 2006a,b,c; Sunagawa et al., 2009; Voolstra et al., 2009; De Salvo et al., 2010; Peng et al., 2010; Ganot et al., 2011; Yuyama et al., 2011; Fransolet et al., 2012; Meyer and Weis, 2012). Adopting an explicitly comparative perspective that unites the findings from different symbioses may elucidate pathways common to intracellularlity *sensu lato*.

## Conclusion

The purpose of this perspective is to focus attention on significant trade-offs that exist for phototrophic symbionts residing in heterotrophic host cells. Constraints exist on investment strategies involving energy that is represented by fixed carbon produced through photosynthesis. The possible phenotypic responses to the trade-offs have significant evolutionary implications. To study these trade-offs, we must understand the cellular environment that the symbionts reside in because important symplesiomorphies likely exist among the various organisms that engage in this type of ecological interaction. One way to achieve success in this area is to increase the dialog that occurs among scientists working with different symbioses. By using names unique to specific hosts to describe endomembranous spaces that phototrophs live in, we may be missing important clues to how symbionts establish stable populations within a particular host. Finally, if we shift attention away from host “control” of the associations, and instead think about the role the symbiont might play in shaping the interactions, we may discover novel theoretical and empirical approaches that have broad explanatory power.

### Conflict of interest statement

The author declares that the research was conducted in the absence of any commercial or financial relationships that could be construed as a potential conflict of interest.
